# Prevalence of monoclonal gammopathy in HIV patients in 2014

**DOI:** 10.7448/IAS.17.4.19649

**Published:** 2014-11-02

**Authors:** Philippe Genet, Laurent Sutton, Driss Chaoui, Ahmad Al Jijakli, Juliette Gerbe, Virginie Masse, Bouchra Wifaq

**Affiliations:** Hematology, Centre Hospitalier Victor Dupouy, Argenteuil, France

## Abstract

**Introduction:**

In non-HIV patients, Monoclonal Gammopathy of Undetermined Significance (MGUS) is associated with an increased risk of subsequent development of haematologic malignancies, especially multiple myeloma (MM) and it has been recently demonstrated that MM is always preceded by a MGUS phase. A higher prevalence of MGUS and MM has been observed in HIV patients compared to the general population. Nevertheless, it has been shown that MGUS in the context of HIV can disappear with antiretroviral therapy (ART). So, measuring MGUS prevalence in HIV patients in the recent period appears of special interest.

**Materials and Methods:**

From January to June 2014, in each out-patient seen in our unit, a serum protein electrophoresis was performed.

**Results:**

A total of 393 patients were screened. Eight patients with HIV2 and one patient with HIV1+HIV2 infection were excluded. Finally, 383 patients (173 female, 210 male) with HIV1 infection were analyzed. Characteristics of patients were as follows: median age 42.2 years (19.1–79.1), hepatitis B virus (HBV) and/or hepatitis C virus (HCV) co-infection 47 (18.8%), median CD4 610 (2–1758), CD8 793 (113–4010), presence of a past AIDS event for 88 patients (23%). Median time with HIV infection was 11 years (0–30). Three hundred fifty-nine patients (93.7%) were on ART for a median duration of 105 months (0–287). For 320 patients (83.6%), viral load was below 50 viral copies/ml. Twelve cases of MGUS (3.1%) were observed: IgG Kappa (five cases), IgG Lambda (five cases), biclonal with two IgG Kappa (one case) and in one case, three monoclonal immunoglobulins were observed (IgG Kappa×2+IgG Lambda). The monoclonal immunoglobulin's level was low and below 1 g/l in all cases except two (2.1 and 11.6 g/l). No factor was found to be predictive of the presence of MGUS in particular age, CD4, HBV/HCV co-infection, viral load or ART.

**Conclusions:**

In the context of modern ART, the prevalence of MGUS remains above those observed in the general population. Even if the level of monoclonal spike observed in our cohort is generally low, an excess risk of subsequent development of MM could be present. Nevertheless, a prospective follow-up of HIV patients with MGUS is necessary to determine this risk.

